# Long noncoding RNAs coordinate functions between mitochondria and the nucleus

**DOI:** 10.1186/s13072-017-0149-x

**Published:** 2017-08-23

**Authors:** Yaru Dong, Takeshi Yoshitomi, Ji-Fan Hu, Jizhe Cui

**Affiliations:** 1 Department of Ophthalmology, The Second Hospital of Jilin University, 218 Ziqiang Street, Changchun, 130041 Jilin China; 2 Stanford University Medical School, VA Palo Alto Health Care System, 3801 Miranda Avenue, Palo Alto, CA 94304 USA; 3 Department of Ophthalmology, Akita University School of Medicine, 1-1-1 Hondo, Akita, 010-8543 Japan; 4 Stem Cell and Cancer Center, First Affiliated Hospital, Jilin University, Changchun, 130061 Jilin China

**Keywords:** Long noncoding RNA, Mitochondria, Nucleus, Coordination, RNA-binding protein

## Abstract

In animal cells, mitochondria are the primary powerhouses and metabolic factories. They also contain genomes and can produce mitochondrial-specific nucleic acids and proteins. To maintain homeostasis of the entire cell, an intense cross-talk between mitochondria and the nucleus, mediated by encoded noncoding RNAs (ncRNAs), as well as proteins, is required. Long ncRNAs (lncRNAs) contain characteristic structures, and they are involved in the regulation of almost every stage of gene expression, as well as being implicated in a variety of disease states, such as cancer. In the coordinated signaling system, several lncRNAs, transcribed in the nucleus but residing in mitochondria, play a key role in regulating mitochondrial functions or dynamics. For example, *RMRP*, a component of the mitochondrial RNase MRP, is important for mitochondrial DNA replication and RNA processing, and the steroid receptor RNA activator, *SRA*, is a key modulator of hormone signaling and is present in both the nucleus and mitochondria. Some RNA-binding proteins maybe play a role in the lncRNAs transport system, such as HuR, GRSF1, SHARP, SLIRP, PPR, and PNPASE. Furthermore, a series of nuclear DNA-encoded lncRNAs were implicated in mitochondria-mediated apoptosis, mitochondrial bioenergetics and biosynthesis, and glutamine metabolism. The mitochondrial genome can also encode a set of lncRNAs, and they are divided into three categories: (1) *lncND5*, *lncND6*, and *lncCyt b* RNA; (2) chimeric mitochondrial DNA-encoded lncRNAs; and (3) putative mitochondrial DNA-encoded lncRNAs. It has been reported that the mitochondrial DNA-encoded lncRNAs appear to operate in the nucleus. The molecular mechanisms underlying trafficking of the mitochondrial DNA-encoded lncRNAs to the nucleus in mammals are only now beginning to emerge. In conclusion, both nuclear- and mitochondrial DNA-encoded lncRNAs mediate an intense intercompartmental cross-talk, which opens a rich field for investigation of the mechanism underlying the intercompartmental coordination and the maintenance of whole cell homeostasis.

## Background

In animal cells, mitochondria also contain genomes, and they can produce mitochondrial-specific nucleic acids and proteins. Because the mitochondria are the primary powerhouses and metabolic factories in the cell, they need to be fully integrated into the whole cell regulation and coordination mechanisms to ensure that cellular needs are met. Thus, the mitochondria must continuously communicate with the nucleus [1]. It is not surprising that an intense cross-talk between mitochondria and the nucleus, mediated by proteins as well as noncoding RNAs (ncRNAs), is required for cellular homeostasis. Long ncRNAs (lncRNAs), which contains characteristic structures, represent a new frontier in the molecular biology of complex organisms, as they participate in the regulation of almost every stage of gene expression, as well as being involved in a variety of disease states [2]. In the coordinated signaling system, several lncRNAs are transcribed in the nucleus but reside in mitochondria and play a key role in regulating mitochondrial functions or dynamics [3]. Some RNA-binding proteins (RBPs) maybe play a role in the lncRNA transport system. Furthermore, a series of nuclear DNA-encoded lncRNAs were implicated in mitochondria-mediated apoptosis, mitochondrial bioenergetics and biosynthesis, and glutamine metabolism. The mitochondrial genomes also encode a set of lncRNAs [4], and they are divided into three categories: (1) *lncND5*, *lncND6*, and *lncCyt b* RNA; (2) chimeric mitochondrial DNA-encoded lncRNAs; and (3) putative mitochondrial DNA-encoded lncRNAs. It has been reported that mitochondrial DNA-encoded lncRNAs seem to operate in the nucleus [5]. The molecular mechanisms underlying trafficking of the mitochondrial-encoded lncRNAs to the nucleus in mammals are only now beginning to emerge.

## Cross-talk between mitochondria and the nucleus

### Mitochondrial endosymbiosis and the mitochondrial genome in humans

Mitochondria are the only organelles in animal cells that possess their own genomes [6]. It is now widely accepted that mitochondria are of endosymbiotic origin, derived from progenitors that resembled extant α-proteobacteria [7, 8]. Following endosymbiosis, the mitochondrial genome has been streamlined into a small, high-copy, bioenergetically specialized genetic system [9].

The human mitochondrial DNA (mtDNA) genome is a compact, circular, double-stranded DNA encoding only 13 proteins, which are all subunits of the electron transport chain, as well as two rRNAs (12S and 16S) and 22 tRNAs required for their translation [10]. In addition to these regions, there is a main control region, or D-loop, which contains the mtDNA replication origin and promoters for mitochondrial RNA (*mtRNA*) transcription [6]. Mitochondrial genes for proteins and tRNAs are located on both strands (historically termed “heavy” and “light” strand) of the mtDNA, which are transcribed as large polycistronic transcripts covering almost the entire length of each strand [4]. *mtRNAs* are processed according to the “transfer RNA (tRNA) punctuation model,” whereby 22 interspersed tRNAs are excised to concomitantly release individual rRNAs and mRNAs [6].

### Mitochondrial function

In classical biochemistry textbooks, the major tasks of the mitochondria are the production of ATP and the metabolites necessary to fulfill the bioenergetic and biosynthetic demands of the cell [1]. Mitochondria use multiple carbon fuels to produce ATP and metabolites, including pyruvate, which is generated from glycolysis; amino acids, such as glutamine; and fatty acids. Mitochondrial respiration accomplishes complete oxidation of substrates, derived from nutrients, to H_2_O and CO_2_ [6]. In addition to generating NADH and FADH_2_, the TCA cycle generates intermediates that can funnel into multiple biosynthetic metabolic pathways to produce glucose, amino acids, lipids, heme, and nucleotides. Thus, the mitochondria operate as a central hub of both catabolic (the breakdown of large macromolecules to produce energy) and anabolic (the production of large macromolecules from small metabolic intermediates using energy) metabolism.

In fact, mitochondrial biological activities have progressively expanded to include not only various bioenergetic processes and important biosynthetic pathways but also calcium homeostasis and thermogenesis, cell death by apoptosis, and several different signal transduction pathways that are primarily related to redox control of gene expression [6]. Oncogenic activation also increases mitochondrial metabolism to generate ATP and TCA cycle intermediates used as precursors for macromolecule synthesis [11]. Currently, the consensus in the cancer metabolism field is that tumor cells robustly engage in both glycolysis and mitochondrial metabolism to provide the necessary building blocks for macromolecule (nucleotides, lipids, and amino acids) synthesis, as well as ATP and NADPH, which are essential for cell proliferation [12]. Altogether, dysregulated mitochondrial function is associated with a variety of physiologic and pathologic processes in the cardiovascular, immune, neurological, and musculoskeletal systems [3].

### Cross-talk between mitochondria and the nucleus

The human mtDNA encodes only 13 proteins of the respiratory chain and ATP synthase complexes; a large proportion of mitochondrial proteins necessary for maintaining mitochondrial structure and function are encoded by the nuclear genome. These nuclear DNA-encoded proteins must be synthesized in the rough endoplasmic reticulum and imported into mitochondria [13, 14]. Protein transport into mitochondria is mediated by four protein complexes that are embedded in the outer and inner membranes of mitochondria: TOM, TIM23, TIM22, and SAM (sorting and assembly machinery) complexes [14, 15]. The central, and the only essential, component of the TOM complex is Tom40, a β-barrel protein which forms a translocation channel [15]. The multiprotein complexes of the mitochondrial respiratory chain are actually mosaics of subunits encoded by nuclear and mitochondrial genes. Mechanisms that coordinate gene expression in the mitochondria and nucleus are required to ensure appropriate and energy-saving assembly of such multiprotein complexes [16].

As mentioned above, the mitochondria have distinct genetic systems that need to be coordinated with the nucleus to ensure cellular demands. Consequently, mechanisms have evolved in which nuclear genes exert direct control of mitochondrial gene expression and posttranslational modifications (anterograde signaling). However, the mitochondrial genome allows individual mitochondria to respond to changes in membrane potential and maintain oxidative phosphorylation by expressing their own genes [9]. This developmental and metabolic information of mitochondria, in turn, is conveyed to the nucleus (retrograde signaling), which enables nuclear gene expression to be modified in accordance with the current status of the organelle [16]. Therefore, it is not surprising that an intense cross-talk between mitochondria and the nucleus, mediated by proteins as well as ncRNAs, is required for cellular homeostasis.

## Long noncoding RNAs

### Noncoding RNAs

Although biologists often speak of a tight coupling between “genes and their encoded protein products,” it is important to remember that thousands of human genes produce ncRNAs as their ultimate products [17]. ncRNAs do not have translated open reading frames [18]. In contrast to the fairly reliable and complete annotation of the protein-coding genes in the human genome, comparable information is lacking for ncRNAs. A large class of ncRNAs has characteristic structures that are functional, and hence, are well conserved over evolutionary timescales. Mapping of conserved RNA secondary structures has predicted that the number of ncRNAs in vertebrate genomes is at least comparable to that of protein-coding genes; there are thousands of functional ncRNAs in the human genome [19].

Based on the length of ncRNAs, regulatory ncRNAs can be divided into at least three groups: (1) short ncRNAs, including microRNA (miRNA) (22–23 nts) and piwi-interacting RNA (piRNA) (26–31 nts); (2) medium ncRNAs (50–200 nts); (3) lncRNAs (>200 nts) [20]. The lncRNAs range from 200 nts to over 100 kb in length, do not contain an open reading frame, and are involved in regulatory functions.

In human cells, ncRNAs originate not only from the nuclear genome but can also be obtained from mtDNA. Both strands of mitochondrial DNA are entirely transcribed, yielding, after processing of the transcripts, a set of ncRNAs [4]. It has been reported that six small ncRNAs have been identified in mitochondria [21], as well as a chimeric lncRNA that contains an inverted repeat of 815 nucleotides covalently linked to the 5′ end of the mitochondrial 16S rRNA synthesized in mitochondria [22].

### Structure of lncRNAs

Certain functional properties of RNA are determined by their primary structure. However, RNA also has a unique ability to adopt a variety of complex secondary and tertiary folds [2]. RNA secondary structures contain single-stranded regions that connect helices, loops, and bulges. These secondary structures dictate the formation of RNA tertiary structure through coaxial stacking of adjacent helices at junctions, pseudoknots, and kissing loops and through networks of triple helices, tetraloop–receptor interactions, and other structures dictated by sequence-specific or backbone interactions [23].

Because of its long length, lncRNA has the potential to develop multiple intramolecular RNA–RNA interactions conferring on them complex three-dimensional structures. It is possible that all lncRNAs consist of multiple structural or functional domains. In addition, in comparison to other classes of RNA, a feature of lncRNA is its propensity to contain a huge number of protein-binding sites. Thus, lncRNA can provide a platform for the multimerization of proteins [24] or can provide a scaffold for the assembly of multiple proteins into larger functional units [25]. It has been demonstrated that many lncRNAs act in conjunction with proteins and other nucleic acids and that their secondary or tertiary structures are important for mediating these interactions [2]. lncRNA and protein complexes function to alter the chromatin state, organize nuclear substructures, and regulate gene expression [2]. For example, the steroid receptor RNA activator (*SRA*) transcript may generate a lncRNA that is the target for a range of RNA-binding nuclear receptor coregulator proteins, including p68, SHARP, and SLIRP, and is a key modulator of hormone signaling [26].

### RNA-binding proteins

RBPs are collectively referred to as heterogeneous ribonucleoproteins that associate with certain transcripts and profoundly influence all steps of posttranscriptional regulation of RNAs, including pre-mRNA splicing and mRNA polyadenylation, stability, localization, translation, and turnover [27–29]. A series of RBPs can mediate RNA transport from the nucleus to mitochondria and vice versa. Although less is known regarding the mechanisms that mediate the lncRNA splicing, stability, transport, and storage, the information emerging on lncRNA-RBP complexes suggests that the same RBPs can bind coding RNA and ncRNA and affect their posttranscriptional fate in various ways [30].

Human antigen R (HuR), a ubiquitous RBP, recognizes specific RNA signature sequences that are typically U- or AU-rich and are usually found in the 3′-untranslated region of short-lived mRNAs [31, 32]. HuR interacts with target RNAs via its three RNA recognition motifs (RRMs). HuR has been shown to stabilize many target mRNAs, but it can also enhance the translation of certain mRNAs (e.g., MKP-1, p53, prothymosin a, and HIF-1a) and repress the translation of other mRNAs (e.g., p27, Wnt5a, and IGF-1R) [32]. Given that HuR is predominantly localized in the nucleus, there has been much interest that HuR can shuttle across the nuclear envelope, in some cases mobilizing target mRNAs (CD83, COX-2) in the process [31, 33].

G-rich RNA sequence-binding factor 1 (GRSF1) is a cytoplasmic RBP and bears three RRMs with high affinity for G-rich sequences [34]. GRSF1 was shown to regulate viral and cellular RNA metabolism at many levels, including splicing, polyadenylation, export, and recruitment to polysomes [35]. An isoform of GRSF1 is targeted to mitochondria where it forms granules that colocalize with foci of newly synthesized *mtRNA* next to mitochondrial nucleoids. GRSF1 preferentially binds RNAs transcribed from three contiguous genes on the light strand of mtDNA, the *ND6 mRNA*, the *lncCyt b* and *lncND5* RNAs, all of which contain the multiple-consensus binding sequence AGGGD. GRSF1 appears to be crucial for posttranscriptional storage, handling, and translation of *mtRNAs* [36].

SMRT/HDAC1-associated repressor protein (SHARP) is an RNA-binding nuclear receptor coregulator protein that includes three RRMs. SHARP is a transcriptional repressor, which can interact with the *SRA*. As mentioned above, *SRA*, a nuclear DNA-encoded lncRNA, has been shown to potentiate steroid hormone receptor transcriptional activity. SHARP binds to the *SRA* via three conserved RRMs and suppresses *SRA* activity. Surprisingly, the expression of SHARP is estrogen inducible, providing a potential autoregulatory mechanism to attenuate the hormonal response [37].

*SRA* stem-loop interacting RNA-binding protein (SLIRP) contains an RNA-binding domain and associates with and represses the activity of *SRA* [38]. However, SLIRP is predominantly localized to the mitochondria [38, 39]. SLIRP plays an essential role in maintaining *mtRNAs* by directly modulating the expression, processing or stability of the mitochondrial transcripts that encode oxidative phosphorylation protein subunits. Furthermore, SLIRP is consistently coexpressed with the nuclear oxidative phosphorylation machinery. These properties raise the interesting possibility that SLIRP is coregulated with mitochondrial and nuclear genes to coordinate the oxidative phosphorylation gene expression of these two genomes [40].

The pentatricopeptide repeat (PPR) protein family is characterized by the presence of a 35-amino-acid structural motif that is tandemly repeated 2–26 times per protein. The PPR domain is involved in RNA–protein interaction, and PPR proteins play various roles in RNA editing, stability, and translation activation [41]. In mammals, seven PPR proteins have been characterized so far [42], and they are mainly localized to mitochondria: POLRMT, pentatricopeptide repeat domain (PTCD) 1, PTCD2, PTCD3, mitochondrial ribosomal protein S27 (MRPS27), leucine-rich pentatricopeptide repeat-containing protein (LRPPRC), and mitochondrial RNase P protein 3 (MRPP3). The LRPPRC is described below. PTCD1 is involved in the processing of mitochondrial polycistronic transcripts that contain leucine tRNA^168^, and PTCD3 is a translational regulator [43]. Knockdown of the PTCD1 protein negatively affected the *lncND5* transcript, whereas impairing PTCD2 decreased the levels of both *lncND5* and *lncND6* RNA [44]. Knockdown of MRPP3 caused a decrease in the abundance of *lncND5*, *lncND6*, and *lncCyt b* RNA; Thus, MRPP3 is necessary for the accumulation of mitochondrial lncRNAs [45].

LRPPRC, an RNA-binding protein, is involved with mtDNA transcript processing. A report found LRPPRC to be bound to polyadenylated mRNA in a shuttling complex with heteronuclear ribonuclear protein K [46], which has been shown to bind mtDNA-encoded mRNA [47]. Furthermore, LRPPRC has been found to be associated with another mitochondrial RBP, SLIRP. LRPPRC and SLIRP are postulated to be part of the high-molecular-mass ribonucleoprotein complex that regulates the metabolism of mitochondrial transcripts [48]. In addition, several reports have shown the presence of LRPPRC in the nucleus, where it seems to play a role in the regulation of mitochondrial biogenesis and energy homeostasis [49], thus suggesting an involvement in the coordination of interactions between the nucleus and mitochondria. Taken together, these findings suggest that LRPPRC participates in the processing and trafficking of mtDNA-encoded RNAs in both the mitochondrion and the nucleus [50].

Polynucleotide phosphorylase (PNPASE) is a 3′- to 5′-exoribonuclease and poly-A polymerase, located in the mitochondrial intermembrane space [51–53]. PNPASE has been recently identified as a novel specific component in the regulation of the import of nuclear-encoded RNAs into the mitochondrial matrix [54]. Mammalian PNPASE may function as an RNA receptor to augment translocation of RNAs [55]. The data from import assays and RNA-binding assays indicated that PNPASE imports RNAs and is dependent on the presence of a specific stem-loop secondary structure in the target RNAs [54, 56].

### Function of lncRNAs

lncRNAs represent a new field of the molecular biology, as it is becoming increasingly apparent that they are involved in the regulation of almost every stage of gene expression, as well as being implicated in a variety of disease states [2]. lncRNAs can regulate chromosomal dynamics, telomere biology, chromatin modification, transcription, posttranscription activity, or metabolic function [57–60]. Transcription of lncRNAs is now known to regulate the expression of protein-coding genes in close genomic proximity (as cis or cis-acting elements) and to target distant transcriptional complexes, such as activators or repressors (in a trans or trans-acting manner), via a variety of mechanisms [22]. Interestingly, a large number of lncRNAs are specifically expressed during embryonic stem cell differentiation, pathogenesis or tumorigenesis [22]. The implications of lncRNAs in tumorigenesis, metastasis, and progression remain to be further investigated. Moreover, increasing evidence suggests that mutation and dysregulation of lncRNAs can result in aberrant expression of gene products that contribute to neurodegenerative disorders [61–64]. However, there is still a large number of lncRNAs for which the biological significance is not yet identified [22].

## Some nuclear DNA-encoded lncRNAs translocate from the nucleus to mitochondria

Mitochondria have distinct genetic systems that need to be coordinated with the nuclear genetic system, to meet the cellular energy and metabolite needs [5]. In the coordinated signaling system, many proteins encoded by nuclear DNA are localized and acted in mitochondria. In addition, some ncRNAs are transcribed in the nucleus but reside in mitochondria and also play a key role in regulating mitochondrial functions [3]; however, the knowledge of the exact mechanism underlying their functions has remained limited. Computational searches for putative genes of mitochondria-localized nuclear-encoded RNAs predicted targeting of a wide range of nuclear genes involved in major mitochondrial functions and in mitochondrial homeostasis [5]. Analysis of the mitochondrial transcriptome led to the systematic identification of several nuclear DNA-encoded lncRNAs present in the mitochondrial inner compartment, the matrix [4]. It has been reported that the nuclear DNA-encoded lncRNA components of RNase P and MRP enzymes are imported into mitochondria and function in mtDNA replication and transcription [65]. The discovery of nuclear DNA-encoded lncRNAs in mitochondria not only provokes challenging questions regarding the role of these molecules in controlling energy homeostasis and metabolism but also leads to questions of how they may coordinate signaling pathways between the nucleus and mitochondria.

Mitochondria possess pathways that enable RNA uptake from the cytosol. Even though the RNA import is a universal process [54, 66], the molecular mechanisms underlying the process are only now beginning to emerge. These nuclear-encoded RNAs in mitochondria have potentially diverse import pathways, and the details of four RNA import mechanisms of mitochondria have been postulated. First, evidence from a study of noncoding 5S rRNA and tRNA targeted to mitochondria suggests an RNA import pathway dependent on the protein import system [67–70]. Second, the RBP of PNPASE, localized in the mitochondrial intermembrane space, facilitates the transfer of a hybrid RNA into the mitochondrial matrix [55]. This methodology is effective for both ncRNAs and mRNAs. Third, translocation of ncRNA could involve voltage-dependent anion channels which located across the outer membrane and mediated inward and outward movements of hydrophilic metabolites [71]. The last hypothesis is that ncRNAs are delivered to mitochondria through a specific system of vesicles [72]. Very few kinds of literature have been reported about the factors that regulate nuclear DNA-encoded lncRNA import into mitochondria.

**Fig. 1:**
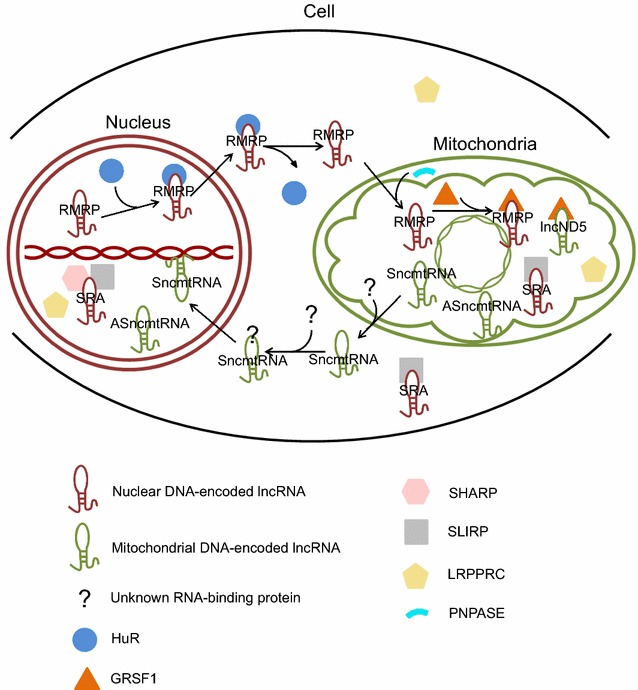
Cross-talk between the nucleus and mitochondria by lncRNAs encoded by each genome for intercompartmental coordination and maintenance of whole cell homeostasis

*RMRP*, a nuclear DNA-encoded lncRNA, is important for mtDNA replication and RNA processing. The gene for *RMRP* is located on chromosome 9p; the full length of *RMRP* is 265 nucleotides in human [73]. Three RBPs (HuR, PNPASE, and GRSF1) are implicated in the transport and localization of *RMRP* from the nucleus to mitochondria (Fig. 1). It has been reported that HuR bound *RMRP* in the nucleus and promoted its export to the cytoplasm [3]. PNPASE, located in the mitochondrial intermembrane space, regulated the import of *RMRP* into the mitochondrial matrix [55]. GRSF1 has been implicated in mitochondrial-related processes, as it was found in submitochondrial domains termed “RNA granules.” After *RMRP* was imported into mitochondria, GRSF1 bound *RMRP* and increased its abundance in the matrix [3]. *RMRP* is the RNA component of the mitochondrial RNA-processing endoribonuclease (RNase MRP) and essential for enzymatic activity. RNase MRP has the capacity to cleave mitochondrial RNA complementary to the light strand of the D-loop at a unique site. This cleavage site is one of the transition sites of primer RNA synthesis to DNA synthesis at the leading-strand origin of mitochondrial DNA replication [73]. Li and colleagues using in situ hybridization analysis showed that *RMRP* is primarily located in the nucleolus, but they have also confirmed the presence of low amount of *RMRP* in mitochondria [74]. It has been believed that a small amount of *RMRP* even at the level of detectability would be sufficient to promote an RNase MRP activity [75].

The steroid receptor RNA activator *SRA*, a lncRNA, is the target for a range of RBPs, including SHARP and SLIRP, and it is a key modulator of hormone signaling [26]. Secondary structure predictions suggest the existence of multiple stem loops within *SRA*. *SRA* acts as an RNA scaffold for other coregulators at the transcription initiation site. SHARP interacts with *SRA* through an RNA-binding domain comprised of three RRMs. These RRMs are required by SHARP to repress *SRA*-augmented estrogen-induced transactivation [37]. SLIRP binds to the complex of *SRA* and SHARP and augments SHARP’s corepressor activity on estrogen signaling. Given SLIRP’s nuclear repressive activities and demonstrated presence in the nucleus, it was surprising that SLIRP is predominantly a mitochondrial protein [76]. *SRA* and SLIRP are present in both the nucleus and mitochondria and may, therefore, be expected to perform tasks in both locations. The transport mechanism of *SRA* and SLIRP from the nucleus to mitochondria remains unknown.

*RNase P* is a lncRNA subunit of ribonuclease P which processes the 5′ leader of precursor tRNA [77]. *RNase P* was partially purified from mammalian mitochondria [78], and there was evidence that *RNase P* is imported into the mitochondrial matrix [3, 4, 54]. However, other studies have reported that human mitochondrial ribonuclease P has evolved to be a protein enzyme that does not require a lncRNA component for catalysis [79, 80]. In all of these controversial data, one season can be the amount of the nuclear DNA-encoded *RNase P* in the mitochondria. If the amount of a lncRNA is low, it is difficult to make a difference between the contamination threshold and a real mitochondrial localization. Furthermore, it cannot be excluded that a “passive” nonspecific process could permit the mitochondrial import of various nucleic acids at a low rate in vivo [81].

## Influence of nuclear DNA-encoded lncRNAs on mitochondrial function

Some nuclear DNA-encoded lncRNA could play a role in mitochondria-mediated apoptosis. *ENSMUST00000136025* is a nearby enhancer-like lncRNA of its neighboring BIM gene; BIM is a pro-apoptotic protein and potentiates mitochondria-dependent apoptosis. After sevoflurane exposure, aberrantly upregulated *ENSMUST00000136025* highly induced overexpression of BIM in sevoflurane-treated samples compared with control samples. BIM eventually promoted sevoflurane-induced hippocampal apoptosis [82]. lncRNA *MEG3* induces renal cell carcinoma (RCC) cells apoptosis by activating the mitochondrial pathway. *MEG3* was evidently downregulated in RCC tissues and RCC cell lines. The apoptosis rate of RCC cells was increased significantly after *MEG3* overexpression. Furthermore, overexpression of *MEG3* could reduce the expression of Bcl-2 and procaspase-9 proteins, enhance the expression of the cleaved caspase-9 protein, and promote the release of cytochrome *c* protein to the cytoplasm [83]. Cardiac apoptosis-related lncRNA (*CARL*) can suppress mitochondrial fission and apoptosis by targeting miR-539 and PHB2 (a subunit of the prohibitin complex). PHB2 has a key role in maintaining the homeostasis of mitochondrial dynamics. PHB2 is negatively regulated by miR-539. *CARL* can directly bind to miR-539 and participates in the regulation of mitochondrial network and apoptosis through the miR-539/PHB2 pathway [84]. Homeobox transcript antisense RNA (*HOTAIR*) induces mitochondria-related apoptosis and inhibits tumor growth in head and neck squamous cell carcinoma (HNSCC) in vitro and in vivo. The mitochondrial membrane potential was changed significantly by *HOTAIR* blockage. Mitochondrial calcium uptake 1-dependent cell death was induced by *HOTAIR* depletion. Mitochondrial-related cell death pathway (Bcl-2, BAX, Caspase-3, cleaved Caspase-3, cytochrome *c*) involved in *HOTAIR*-dependent apoptosis process. *HOTAIR* plays a vital role in cancer initiation and progression by affecting cell cycle progress, apoptosis, and invasion [85].

Evidence that several nuclear DNA-encoded lncRNAs may regulate mitochondrial bioenergetics and biosynthesis has also been reported. Taurine upregulated gene 1 (*Tug1*), an evolutionarily conserved lncRNA, regulates mitochondrial bioenergetics in diabetic nephropathy (DN). The PGC-1α (encoded by Ppargc1a), whose expression is typically reduced in diabetes, is a key mediator of mitochondrial dysfunction and progression of DN. *Tug1* is a regulator of PGC-1α transcription. *Tug1* interacts with PGC-1α at its promoter, resulting in elevated Ppargc1a transcriptional output. A decrease in *Tug1* in the diabetic milieu would decrease PGC-1α expression, resulting in decreased expression of downstream PGC-1α targets involved in regulating mitochondrial bioenergetics [86]. *SAMMSON*, a recently annotated lncRNA gene, is located in the chromosome 3p melanoma-specific focal. *SAMMSON* interacts with p32, a master regulator of mitochondrial homeostasis and metabolism, to increase its mitochondrial targeting and pro-oncogenic function. Silencing of the lineage addiction oncogene *SAMMSON* caused mitochondrial protein synthesis defects [87]. lncRNA *AK055347* may contribute to the pathogenesis of atrial fibrillation by dysregulating mitochondrial energy production via regulation of Cyp450, ATP synthase, and MSS51 [88].

A series of lncRNAs was implicated in glutamine metabolism. Glutamine, one of the essential nutrients, is deaminated by glutaminase (GLS) to produce glutamate, which further serves as a substrate for the mitochondrial tricarboxylic cycle [89]. lncRNA *HOTTIP* is a vital oncogenesis factor, significantly upregulated in hepatocellular carcinoma specimens [90]. GLS was identified as a potential downstream target of *HOTTIP* [91]. The posttranscriptional silencing of *HOTTIP* could significantly suppress viability of hepatocellular carcinoma cells. Furthermore, the lncRNA *CCAT2* is specifically overexpressed in microsatellite stable colorectal cancer and regulates the alternative splicing of GLS in an allele-specific manner [89]. Moreover, in bladder cancer cells, the lncRNA *UCA1* regulated the expression of GLS by interfering with miR-16 and repressed reactive oxygen species (ROS) formation [92]. ROS are the most important byproducts of the electron transport chain in the mitochondria. Increased production of ROS can lead to activation of tumorigenic signaling and metabolic reprogramming [93].

## Mitochondrial DNA-encoded lncRNAs

### Classification of mitochondrial DNA-encoded lncRNAs

In animals, after processing the polycistronic transcripts of mtDNA, a set of ncRNAs was found [4]. The mitochondrial polycistronic transcript encoding the heavy-strand genes has the little noncoding sequence. In contrast, the light-strand polycistronic transcript only encodes seven tRNAs and the ND6 mRNA, which are separated by long stretches of noncoding sequences [45]. Little is known regarding the presence of the mitochondrial-encoded ncRNAs. It has been reported that lncRNAs mapping to the mtDNA seem to operate in the nucleus. The trafficking of mitochondrial DNA-encoded lncRNAs to the nucleus in mammals raises questions far beyond the current knowledge [5]. lncRNAs transcribed from the mtDNA are divided into three categories: (1) *lncND5*, *lncND6,* and *lncCyt b* RNA; (2) chimeric mitochondrial DNA-encoded lncRNAs; (3) putative mitochondrial DNA-encoded lncRNAs.

### lncND5, lncND6, and lncCyt b

Three abundant mitochondrial DNA-encoded lncRNAs, *lncND5*, *lncND6*, and *lncCyt b* RNA, have been identified in humans. The regions of the mitochondrial genome complementary to the genes that encode ND5, *ND6*, and *Cyt b mRNAs* were found to have high levels of lncRNAs, specifically, *lncND5*, *lncND6*, and *lncCyt b* RNA. These three lncRNAs are 58, 34, and 14% as abundant as their complementary coding *ND5*, *ND6*, and *Cyt b mRNAs*, respectively. These mitochondrial DNA-encoded lncRNAs primarily form intermolecular duplexes with their respective complementary mRNAs. This suggests that they may have a functional role in mitochondria to either stabilize their partner mRNAs or regulate their expression. *lncND5* RNA was the most abundant of the three mitochondrial DNA-encoded lncRNAs. The expression of *lncND5*, *lncND6*, and *lncCyt b* RNA is regulated by nuclear-encoded mitochondrial processing proteins, such as the PPR protein family [45]. It was speculated that a putative role of such mitochondrial DNA-encoded lncRNAs could be coordination of the nuclear and mitochondrial transcripts.

### Chimeric mitochondrial DNA-encoded lncRNAs

A set of chimeric mitochondrial DNA-encoded lncRNAs with surprising structures and behavior was identified in mice and humans. The first member (1685 nucleotides) includes the mitochondrial 16S rRNA, together with a 121 nucleotide 5′-leader sequence deriving from the complementary strand of the gene encoding this rRNA [94]. A second transcript (2374 nucleotides) of a similar type, called sense mitochondrial ncRNA (*SncmtRNA*), was identified in proliferating human tumor cells [21]. In *SncmtRNA*, the mitochondrial 16S rRNA was associated with an 815 nucleotide 5′-leader fragment deriving from the complementary strand. The *SncmtRNA* was polyadenylated and formed an 820 bp, RNase-resistant double-stranded structure with a 40 nucleotide loop [5]. Subsequent analyses in normal proliferating cells further revealed two antisense mitochondrial DNA-encoded lncRNAs, called *ASncmtRNA*-*1* and *ASncmtRNA*-*2*, combining the antisense mitochondrial 16S rRNA with a 310 or 545 nucleotide 5′-leader sequence deriving from the sense strand of the gene encoding this rRNA [95]. These two *ASncmtRNAs* formed distinct double-stranded structures of 310 or 545 bp with a 96 or 450 nucleotide loop, respectively.

The *SncmtRNA* and *ASncmtRNAs* were reported to be present in both the mitochondria and the nucleus, suggesting a role in mitochondrial nuclear communication and retrograde signaling [96]. Furthermore, these data imply an impressive in- and out-organellar trafficking of these lncRNAs. There were clearly missing data regarding the mechanisms able to support the putative trafficking of such large and structured RNAs. *ASncmtRNA*-*2* has been shown to be exported from the mitochondria into the nucleus [96]. It was postulated that *ASncmtRNA*-*2* might function as a precursor of two miRNAs (hsa-miR-4485 and hsa-miR-1973) [97, 98], but its transport mechanisms are still largely unknown. Possible roles of the mitochondrial DNA-encoded lncRNAs in intercompartmental cross-talk and genetic regulation underlying cellular homeostasis are emerging, opening a rich field of investigation.

Recently, it has been postulated that the *ASncmtRNAs* might function as unique tumor suppressors [97]. Although normal proliferating cells express *SncmtRNA* and *ASncmtRNAs*, the *ASncmtRNAs* are downregulated in human tumor cell lines and tumor cells from biopsies of different types of cancer, and they may take part in the mitochondrial reprogramming of oncogenic pathways [95]. Knockdown of *ASncmtRNAs* potentiates apoptotic cell death by inhibiting survivin expression, a member of the inhibitor of apoptosis family. Downregulation of survivin is at the translational level and is probably mediated by miRNAs generated by dicing of the double-stranded stem of the *ASncmtRNAs* [97].

### Putative mitochondrial DNA-encoded lncRNAs

A series of putative mitochondrial DNA-encoded lncRNAs was identified in studies devoted to heart diseases [5, 99, 100]. Deep sequencing revealed a high relative abundance (over 70%) of the putative mitochondrial DNA-encoded lncRNAs in the total lncRNA population from patients with severe heart failure compared to non-failing human samples [99]. Among these putative mitochondrial DNA-encoded lncRNAs, the most significant was called long intergenic noncoding RNA predicting CARdiac remodeling (*LIPCAR*). *LIPCAR*, a chimeric lncRNA, contains 781 nucleotides. Mapping the complete *LIPCAR* sequence to the human mitochondrial genome showed that the 5′ half (nucleotides 1–392) maps to antisense of the mitochondrial CYTB gene, but the 3′ half (nucleotides 385–781) maps to antisense of the mitochondrial COX2 gene. Indeed, the 5′ half of *LIPCAR* is wholly contained within the previously described mitochondrial *lncCyt b* gene [101]. The level of circulating *LIPCAR* was upregulated at the late stages of left ventricular remodeling and was elevated in patients with chronic heart failure, independently of the pathogenesis. Thus, this lncRNA behaved as a prognostic indicator for chronic heart failure and cardiovascular mortality [5].

## Conclusion

LncRNAs are emerging as new players in gene regulation, but how lncRNAs operate in the regulation of an intense cross-talk between nucleus and mitochondria is still unclear. Because mitochondrial biological activities include not only bioenergetic processes and biosynthetic pathways but also calcium homeostasis, thermogenesis, and cell apoptosis [6], the function and underlying mechanism of the lncRNAs implicated in the coordinating system are likely very complex. It is an exciting time to begin thinking about the lncRNAs targeting intercompartmental coordination. Several fundamental questions should be addressed. First, a crucial issue is the investigation of the molecular mechanisms by which trafficking of the nuclear- or mitochondrial DNA-encoded lncRNAs between the two organelles is accomplished. Two related questions should also be asked: what RBPs are bound, and how do these lncRNAs go through the nuclear envelope or mitochondrial membrane? Second, what are the target genes of these lncRNAs, and how are they regulated? Finally, is the intercompartmental coordinating mechanism of lncRNAs changed in disease? After uncovering these questions, better knowledge regarding lncRNA coordination of the nuclear and mitochondrial function is likely to have important implications for diagnosis and therapy of major mitochondrial-related diseases.

## Abbreviations

ncRNAs: noncoding RNAs
lncRNAs: long ncRNAs
RBPs: RNA-binding proteins
mtDNA: mitochondrial DNA
mtRNA: mitochondrial RNA
tRNAs: transfer RNAs
rRNAs: ribosomal RNAs
snoRNAs: small nucleolar RNAs
snRNAs: small nuclear RNAs
miRNA: microRNA
piRNA: piwi-interacting RNA
SRA: steroid receptor RNA activator
HuR: human antigen R
RRMs: RNA recognition motifs
GRSF1: G-rich RNA sequence-binding factor 1
SHARP: SMRT/HDAC1-associated repressor protein
SLIRP: SRA stem-loop interacting RNA-binding protein
PPR: pentatricopeptide repeat
PTCD1: pentatricopeptide repeat domain 1
MRPS27: mitochondrial ribosomal protein S27
LRPPRC: leucine-rich pentatricopeptide repeat-containing protein
MRPP3: mitochondrial RNase P protein 3
PNPASE: polynucleotide phosphorylase
GLS: glutaminase
ROS: reactive oxygen species
LIPCAR: long intergenic noncoding RNA predicting CARdiac remodeling

## References

[1] Weinberg SE, Chandel NS (2015). Targeting mitochondria metabolism for cancer therapy. Nat Chem Biol.

[2] Blythe AJ, Fox AH, Bond CS (2016). The ins and outs of lncRNA structure: how, why and what comes next?. Biochim Biophys Acta.

[3] Noh JH, Kim KM, Abdelmohsen K, Yoon JH, Panda AC, Munk R (2016). HuR and GRSF1 modulate the nuclear export and mitochondrial localization of the lncRNA RMRP. Genes Dev.

[4] Mercer TR, Neph S, Dinger ME, Crawford J, Smith MA, Shearwood AM (2011). The human mitochondrial transcriptome. Cell.

[5] Dietrich A, Wallet C, Iqbal RK, Gualberto JM, Lotfi F (2015). Organellar non-coding RNAs: emerging regulation mechanisms. Biochimie.

[6] Papa S, Martino PL, Capitanio G, Gaballo A, De Rasmo D, Signorile A (2012). The oxidative phosphorylation system in mammalian mitochondria. Adv Exp Med Biol.

[7] Andersson SG, Karlberg O, Canbäck B, Kurland CG (2003). On the origin of mitochondria: a genomics perspective. Philos Trans R Soc Lond B Biol Sci.

[8] Gray MW, Burger G, Lang BF (1999). Mitochondrial evolution. Science.

[9] Lane N, Martin W (2010). The energetics of genome complexity. Nature.

[10] Smeitink J, van den Heuvel L, DiMauro S (2001). The genetics and pathology of oxidative phosphorylation. Nat Rev Genet.

[11] Dang CV (2012). Links between metabolism and cancer. Genes Dev.

[12] Ward PS, Thompson CB (2012). Metabolic reprogramming: a cancer hallmark even warburg did not anticipate. Cancer Cell.

[13] Pfanner N, Craig EA, Hönlinger A (1997). Mitochondrial preprotein translocase. Annu Rev Cell Dev Biol.

[14] Neupert W, Herrmann JM (2007). Translocation of proteins into mitochondria. Annu Rev Biochem.

[15] Mokranjac D, Neupert W (2009). Thirty years of protein translocation into mitochondria: unexpectedly complex and still puzzling. Biochim Biophys Acta.

[16] Leister D, Kleine T (2011). Role of intercompartmental DNA transfer in producing genetic diversity. Int Rev Cell Mol Biol.

[17] Eddy SR (1999). Noncoding RNA genes. Curr Opin Genet Dev.

[18] Lander ES, Linton LM, Birren B, Nusbaum C, Zody MC, Baldwin J (2001). Initial sequencing and analysis of the human genome. Nature.

[19] Washietl S, Hofacker IL, Lukasser M, Hüttenhofer A, Stadler PF (2005). Mapping of conserved RNA secondary structures predicts thousands of functional noncoding RNAs in the human genome. Nat Biotechnol.

[20] Nie L, Wu HJ, Hsu JM, Chang SS, Labaff AM, Li CW (2012). Long non-coding RNAs: versatile master regulators of gene expression and crucial players in cancer. Am J Transl Res..

[21] Lung B, Zemann A, Madej MJ, Schuelke M, Techritz S, Ruf S (2006). Identification of small non-coding RNAs from mitochondria and chloroplasts. Nucleic Acids Res.

[22] Villegas J, Burzio V, Villota C, Landerer E, Martinez R, Santander M (2007). Expression of a novel non-coding mitochondrial RNA in human proliferating cells. Nucleic Acids Res.

[23] Butcher SE, Pyle AM (2011). The molecular interactions that stabilize RNA tertiary structure: RNA motifs, patterns, and networks. Acc Chem Res.

[24] Duszczyk MM, Wutz A, Rybin V, Sattler M (2011). The Xist RNA A-repeat comprises a novel AUCG tetraloop fold and a platform for multimerization. RNA.

[25] Clemson CM, Hutchinson JN, Sara SA, Ensminger AW, Fox AH, Chess A (2009). An architectural role for a nuclear noncoding RNA: NEAT1 RNA is essential for the structure of paraspeckles. Mol Cell.

[26] Colley SM, Iyer KR, Leedman PJ (2008). The RNA coregulator SRA, its binding proteins and nuclear receptor signaling activity. IUBMB Life.

[27] Dreyfuss G, Kim VN, Kataoka N (2002). Messenger-RNA-binding proteins and the messages they carry. Nat Rev Mol Cell Biol.

[28] Moore MJ (2005). From birth to death: the complex lives of eukaryotic mRNAs. Science.

[29] Keene JD (2007). RNA regulons: coordination of post-transcriptional events. Nat Rev Genet.

[30] Li JH, Liu S, Zheng LL, Wu J, Sun WJ, Wang ZL (2015). Discovery of protein-lncRNA interactions by integrating large-scale CLIP-Seq and RNA-Seq datasets. Front Bioeng Biotechnol.

[31] Doller A, Akool el-S, Huwiler A, Müller R, Radeke HH, Pfeilschifter J (2008). Posttranslational modification of the AU-rich element binding protein HuR by protein kinase Cdelta elicits angiotensin II-induced stabilization and nuclear export of cyclooxygenase 2 mRNA. Mol Cell Biol.

[32] Yi J, Chang N, Liu X, Guo G, Xue L, Tong T (2010). Reduced nuclear export of HuR mRNA by HuR is linked to the loss of HuR in replicative senescence. Nucleic Acids Res.

[33] Fries B, Heukeshoven J, Hauber I, Grüttner C, Stocking C, Kehlenbach RH (2007). Analysis of nucleocytoplasmic trafficking of the HuR ligand APRIL and its influence on CD83 expression. J Biol Chem.

[34] Qian Z, Wilusz J (1994). GRSF-1: a poly(A) + mRNA binding protein which interacts with a conserved G-rich element. Nucleic Acids Res.

[35] Ufer C (2012). The biology of the RNA binding protein guanine-rich sequence binding factor 1. Curr Protein Pept Sci.

[36] Antonicka H, Sasarman F, Nishimura T, Paupe V, Shoubridge EA (2013). The mitochondrial RNA-binding protein GRSF1 localizes to RNA granules and is required for posttranscriptional mitochondrial gene expression. Cell Metab.

[37] Shi Y, Downes M, Xie W, Kao HY, Ordentlich P, Tsai CC (2001). Sharp, an inducible cofactor that integrates nuclear receptor repression and activation. Genes Dev.

[38] Hatchell EC, Colley SM, Beveridge DJ, Epis MR, Stuart LM, Giles KM (2006). SLIRP, a small SRA binding protein, is a nuclear receptor corepressor. Mol Cell.

[39] Pagliarini DJ, Calvo SE, Chang B, Sheth SA, Vafai SB, Ong SE (2008). A mitochondrial protein compendium elucidates complex I disease biology. Cell.

[40] Baughman JM, Nilsson R, Gohil VM, Arlow DH, Gauhar Z, Mootha VK (2009). A computational screen for regulators of oxidative phosphorylation implicates SLIRP in mitochondrial RNA homeostasis. PLoS Genet.

[41] Schmitz-Linneweber C, Small I (2008). Pentatricopeptide repeat proteins: a socket set for organelle gene expression. Trends Plant Sci.

[42] Rackham O, Filipovska A (2012). The role of mammalian PPR domain proteins in the regulation of mitochondrial gene expression. Biochim Biophys Acta.

[43] De Silva Dasmanthie, Ya-Ting Tu, Amunts Alexey, Fontanesi Flavia, Barrientos Antoni (2015). Mitochondrial ribosome assembly in health and disease. Cell Cycle.

[44] Lightowlers RN, Chrzanowska-Lightowlers ZM (2008). PPR (pentatricopeptide repeat) proteins in mammals: important aids to mitochondrial gene expression. Biochem J.

[45] Rackham O, Shearwood AM, Mercer TR, Davies SM, Mattick JS, Filipovska A (2011). Long noncoding RNAs are generated from the mitochondrial genome and regulated by nuclear-encoded proteins. RNA.

[46] Mili S, Shu HJ, Zhao Y, Piñol-Roma S (2001). Distinct RNP complexes of shuttling hnRNP proteins with pre-mRNA and mRNA: candidate intermediates in formation and export of mRNA. Mol Cell Biol.

[47] Ostrowski J, Wyrwicz L, Rychlewski L, Bomsztyk K (2002). Heterogeneous nuclear ribonucleoprotein K protein associates with multiple mitochondrial transcripts within the organelle. J Biol Chem.

[48] Sasarman F, Brunel-Guitton C, Antonicka H, Wai T (2010). Shoubridge EA; LSFC Consortium. LRPPRC and SLIRP interact in a ribonucleoprotein complex that regulates posttranscriptional gene expression in mitochondria. Mol Biol Cell.

[49] Cooper MP, Qu L, Rohas LM, Lin J, Yang W, Erdjument-Bromage H (2006). Defects in energy homeostasis in Leigh syndrome French Canadian variant through PGC-1alpha/LRP130 complex. Genes Dev.

[50] Mootha VK, Lepage P, Miller K, Bunkenborg J, Reich M, Hjerrild M (2003). Identification of a gene causing human cytochrome *c* oxidase deficiency by integrative genomics. Proc Natl Acad Sci USA.

[51] Rainey RN, Glavin JD, Chen HW, French SW, Teitell MA, Koehler CM (2006). A new function in translocation for the mitochondrial i-AAA protease Yme1: import of polynucleotide phosphorylase into the intermembrane space. Mol Cell Biol.

[52] Chen HW, Rainey RN, Balatoni CE, Dawson DW, Troke JJ, Wasiak S (2006). Mammalian polynucleotide phosphorylase is an intermembrane space RNase that maintains mitochondrial homeostasis. Mol Cell Biol.

[53] Chen HW, Koehler CM, Teitell MA (2007). Human polynucleotide phosphorylase: location matters. Trends Cell Biol.

[54] Wang G, Chen HW, Oktay Y, Zhang J, Allen EL, Smith GM (2010). PNPASE regulates RNA import into mitochondria. Cell.

[55] Wang G, Shimada E, Koehler CM, Teitell MA (2012). PNPASE and RNA trafficking into mitochondria. Biochim Biophys Acta.

[56] Lin CL, Wang YT, Yang WZ, Hsiao YY, Yuan HS (2012). Crystal structure of human polynucleotide phosphorylase: insights into its domain function in RNA binding and degradation. Nucleic Acids Res.

[57] Keller C, Bühler M (2013). Chromatin-associated ncRNA activities. Chromosome Res.

[58] Kornfeld JW, Brüning JC (2014). Regulation of metabolism by long, non-coding RNAs. Front Genet..

[59] Krishnan J, Mishra RK (2014). Emerging trends of long non-coding RNAs in gene activation. FEBS J.

[60] Mercer TR, Dinger ME, Mattick JS (2009). Long non-coding RNAs: insights into functions. Nat Rev Genet.

[61] Faghihi MA, Modarresi F, Khalil AM, Wood DE, Sahagan BG, Morgan TE (2008). Expression of a noncoding RNA is elevated in Alzheimer’s disease and drives rapid feed-forward regulation of beta-secretase. Nat Med.

[62] Arisi I, D’Onofrio M, Brandi R, Felsani A, Capsoni S, Drovandi G (2011). Gene expression biomarkers in the brain of a mouse model for Alzheimer’s disease: mining of microarray data by logic classification and feature selection. J Alzheimers Dis.

[63] Johnson R (2012). Long non-coding RNAs in Huntington’s disease neurodegeneration. Neurobiol Dis.

[64] Qureshi IA, Mehler MF (2013). Long non-coding RNAs: novel targets for nervous system disease diagnosis and therapy. Neurotherapeutics..

[65] Bonawitz ND, Clayton DA, Shadel GS (2006). Initiation and beyond: multiple functions of the human mitochondrial transcription machinery. Mol Cell.

[66] Alfonzo JD, Söll D (2009). Mitochondrial tRNA import—the challenge to understand has just begun. Biol Chem.

[67] Kolesnikova OA, Entelis NS, Mireau H, Fox TD, Martin RP, Tarassov IA (2000). Suppression of mutations in mitochondrial DNA by tRNAs imported from the cytoplasm. Science.

[68] Entelis NS, Kolesnikova OA, Dogan S, Martin RP, Tarassov IA (2001). 5 S rRNA and tRNA import into human mitochondria. Comparison of in vitro requirements. J Biol Chem.

[69] Smirnov A, Comte C, Mager-Heckel AM, Addis V, Krasheninnikov IA, Martin RP (2010). Mitochondrial enzyme rhodanese is essential for 5 S ribosomal RNA import into human mitochondria. J Biol Chem.

[70] Smirnov A, Entelis N, Martin RP, Tarassov I (2011). Biological significance of 5S rRNA import into human mitochondria: role of ribosomal protein MRP-L18. Genes Dev.

[71] Salinas T, Duchêne AM, Delage L, Nilsson S, Glaser E, Zaepfel M (2006). The voltage-dependent anion channel, a major component of the tRNA import machinery in plant mitochondria. Proc Natl Acad Sci USA.

[72] Bandiera S, Matégot R, Girard M, Demongeot J, Henrion-Caude A (2013). MitomiRs delineating the intracellular localization of microRNAs at mitochondria. Free Radic Biol Med.

[73] Hsieh CL, Donlon TA, Darras BT, Chang DD, Topper JN, Clayton DA (1990). The gene for the RNA component of the mitochondrial RNA-processing endoribonuclease is located on human chromosome 9p and on mouse chromosome 4. Genomics.

[74] Li K, Smagula CS, Parsons WJ, Richardson JA, Gonzalez M, Hagler HK (1994). Subcellular partitioning of MRP RNA assessed by ultrastructural and biochemical analysis. J Cell Biol.

[75] Topper JN, Bennett JL, Clayton DA (1992). A role for RNAase MRP in mitochondrial RNA processing. Cell.

[76] Colley SM, Leedman PJ (2009). SRA and its binding partners: an expanding role for RNA-binding coregulators in nuclear receptor-mediated gene regulation. Crit Rev Biochem Mol Biol.

[77] Jacobson MR, Cao LG, Taneja K, Singer RH, Wang YL, Pederson T (1997). Nuclear domains of the RNA subunit of RNase P. J Cell Sci.

[78] Doersen CJ, Guerrier-Takada C, Altman S, Attardi G (1985). Characterization of an RNase P activity from HeLa cell mitochondria. Comparison with the cytosol RNase P activity. J Biol Chem.

[79] Rossmanith W, Karwan RM (1998). Characterization of human mitochondrial RNase P: novel aspects in tRNA processing. Biochem Biophys Res Commun.

[80] Holzmann J, Frank P, Löffler E, Bennett KL, Gerner C, Rossmanith W (2008). RNase P without RNA: identification and functional reconstitution of the human mitochondrial tRNA processing enzyme. Cell.

[81] Sieber F, Duchêne AM, Maréchal-Drouard L (2011). Mitochondrial RNA import: from diversity of natural mechanisms to potential applications. Int Rev Cell Mol Biol..

[82] Chen X, Zhou X, Lu D, Yang X, Zhou Z, Chen X (2016). Aberrantly expressed long noncoding RNAs are involved in sevoflurane-induced developing hippocampal neuronal apoptosis: a microarray related study. Metab Brain Dis.

[83] Wang M, Huang T, Luo G, Huang C, Xiao XY, Wang L (2015). Long non-coding RNA MEG3 induces renal cell carcinoma cells apoptosis by activating the mitochondrial pathway. J Huazhong Univ Sci Technol Med Sci.

[84] Wang K, Long B, Zhou LY, Liu F, Zhou QY, Liu CY (2014). CARL lncRNA inhibits anoxia-induced mitochondrial fission and apoptosis in cardiomyocytes by impairing miR-539-dependent PHB2 downregulation. Nat Commun.

[85] Kong L, Zhou X, Wu Y, Wang Y, Chen L, Li P (2015). Targeting HOTAIR induces mitochondria related apoptosis and inhibits tumor growth in head and neck squamous cell carcinoma in vitro and in vivo. Curr Mol Med.

[86] Long J, Badal SS, Ye Z, Wang Y, Ayanga BA, Galvan DL (2016). Long noncoding RNA Tug1 regulates mitochondrial bioenergetics in diabetic nephropathy. J Clin Investig.

[87] Leucci E, Vendramin R, Spinazzi M, Laurette P, Fiers M, Wouters J (2016). Melanoma addiction to the long non-coding RNA SAMMSON. Nature.

[88] Chen G, Guo H, Song Y, Chang H, Wang S, Zhang M (2016). Long non-coding RNA AK055347 is upregulated in patients with atrial fibrillation and regulates mitochondrial energy production in myocardiocytes. Mol Med Rep..

[89] Redis RS, Vela LE, Lu W, Ferreira de Oliveira J, Ivan C, Rodriguez-Aguayo C (2016). Allele-specific reprogramming of cancer metabolism by the long non-coding RNA CCAT2. Mol Cell.

[90] Quagliata L, Matter MS, Piscuoglio S, Arabi L, Ruiz C, Procino A (2014). Long noncoding RNA HOTTIP/HOXA13 expression is associated with disease progression and predicts outcome in hepatocellular carcinoma patients. Hepatology.

[91] Ge Y, Yan X, Jin Y, Yang X, Yu X, Zhou L (2015). MiRNA-192 [corrected] and miRNA-204 directly suppress lncRNA HOTTIP and interrupt GLS1-mediated glutaminolysis in hepatocellular carcinoma. PLoS Genet.

[92] Li HJ, Li X, Pang H, Pan JJ, Xie XJ, Chen W (2015). Long non-coding RNA UCA1 promotes glutamine metabolism by targeting miR-16 in human bladder cancer. Jpn J Clin Oncol.

[93] Beltrán-Anaya FO, Cedro-Tanda A, Hidalgo-Miranda A, Romero-Cordoba SL (2016). Insights into the regulatory role of non-coding RNAs in cancer metabolism. Front Physiol.

[94] Villegas J, Zárraga AM, Muller I, Montecinos L, Werner E, Brito M (2000). A novel chimeric mitochondrial RNA localized in the nucleus of mouse sperm. DNA Cell Biol.

[95] Burzio VA, Villota C, Villegas J, Landerer E, Boccardo E, Villa LL (2009). Expression of a family of noncoding mitochondrial RNAs distinguishes normal from cancer cells. Proc Natl Acad Sci USA.

[96] Landerer E, Villegas J, Burzio VA, Oliveira L, Villota C, Lopez C (2011). Nuclear localization of the mitochondrial ncRNAs in normal and cancer cells. Cell Oncol (Dordr).

[97] Vidaurre S, Fitzpatrick C, Burzio VA, Briones M, Villota C, Villegas J (2014). Down-regulation of the antisense mitochondrial non-coding RNAs (ncRNAs) is a unique vulnerability of cancer cells and a potential target for cancer therapy. J Biol Chem.

[98] Bianchessi V, Badi I, Bertolotti M, Nigro P, D’Alessandra Y, Capogrossi MC (2015). The mitochondrial lncRNA ASncmtRNA-2 is induced in aging and replicative senescence in endothelial cells. J Mol Cell Cardiol.

[99] Yang KC, Yamada KA, Patel AY, Topkara VK, George I, Cheema FH (2014). Deep RNA sequencing reveals dynamic regulation of myocardial noncoding RNAs in failing human heart and remodeling with mechanical circulatory support. Circulation.

[100] Kumarswamy R, Bauters C, Volkmann I, Maury F, Fetisch J, Holzmann A (2014). Circulating long noncoding RNA, LIPCAR, predicts survival in patients with heart failure. Circ Res.

[101] Dorn GW (2014). LIPCAR: a mitochondrial lnc in the noncoding RNA chain?. Circ Res.

